# Expression of the global regulator SATB1 is an independent factor of poor prognosis in high grade epithelial ovarian cancer

**DOI:** 10.1186/1757-2215-5-24

**Published:** 2012-09-19

**Authors:** Björn Nodin, Charlotta Hedner, Mathias Uhlén, Karin Jirström

**Affiliations:** 1 Department of Clinical Sciences, Division of Pathology, Lund University, Lund, SE-221 85, Sweden; 2 Department of Pathology, University and Regional Laboratories Region Skåne, Lund, SE-221 85, Sweden; 3 Department of Proteomics, Royal Institute of Technology, AlbaNova University Center, Stockholm, SE-106 91, Sweden; 4 Science for Life Laboratory, Royal Institute of Technology, Stockholm, SE-106 91, Sweden

**Keywords:** SATB1, Immunohistochemistry, Epithelial ovarian cancer, Prognosis

## Abstract

**Background:**

The global gene regulator Special AT-rich sequence-binding protein1 (SATB1) has been reported to reprogramme tumour cells into a more malignant phenotype and associate with poor clinical outcome in several cancer forms. In this study, we investigated the molecular correlates and prognostic impact of SATB1 expression in human epithelial ovarian cancer (EOC).

**Findings:**

Immunohistochemical expression of SATB1 was examined in tissue microarrays with tumours from 151 incident EOC cases from two prospective, population-based cohorts. Benign-appearing fallopian tube epithelium from 32 cases was also analyzed. A multiplier of nuclear fraction and staining intensity of SATB1 was calculated. While barely expressed in tubal epithelium, nuclear SATB1 expression was denoted in 35/151 (23.2%) EOC cases. Spearman´s Rho test revealed an inverse correlation between SATB1 expression and histological grade (R = -0.22, p = 0.006) and a positive correlation with expression of dachshund 2 protein (R = 0.28, p = 0.001), phosphorylated Chek1 (R = 0.26, p = 0.002) and minichromosome maintenance protein 3 (R = 0.17, p = 0.042). Univariable Cox regression analysis revealed that SATB1 expression, while not prognostic in the full cohort, was associated with a reduced ovarian cancer-specific survival and 5-year overall survival in high grade tumours (n = 105) (HR = 2.14 and HR = 1.96, respectively). This association remained significant in multivariable analysis, adjusted for age and clinical stage (HR = 2.20 and HR = 2.06, respectively).

**Conclusions:**

These results demonstrate that SATB1 expression is an independent factor of poor prognosis in high grade EOC and correlates *in vivo* with cellular processes involved in the maintenance of DNA integrity. The functional basis for these observations merits further investigation.

## Findings

### Background

The T-lineage enriched global chromatin organizer and epigenetic regulator Special AT-rich sequence-binding protein 1(SATB1) [1,2] has been reported to promote a metastatic phenotype and correlate with poor prognosis in breast cancer [3]. SATB1 expression has also been associated with unfavourable clinicopathological characteristics and poor prognosis in gastric, liver and colorectal cancer, and glioma [4-9]. In a recent study on epithelial ovarian cancer (EOC), SATB1 expression was found to be up-regulated both at the mRNA and protein level in EOC (n = 91) compared to borderline tumours and normal ovarian tissue [10]. High SATB1 expression was also found to correlate with increased FIGO stage, lymph node metastasis and reduced overall survival, but it was not reported whether SATB1 was an independent prognostic factor [10]. In the present study, immunohistochemical SATB1 expression was examined in primary tumours from 151 incident cases of EOC from two Swedish population-based cohort studies, and correlated with clinicopathological factors, molecular parameters, and survival. A subset of concomitantly sampled benign-appearing fallopian tubes (n = 32) was also analyzed for SATB1 expression.

### Patients and methods

The study cohort is a merge of incident cases of epithelial ovarian cancers in the Malmö Diet and Cancer Study and Malmö Preventive Project up until 31 Dec 2007, as previously described [11-15]. Information on vital status and cause of death was obtained from the Swedish Cause of Death Registry up until 30 June 2012. After a median follow-up of 3.00 years (range 0–24.63), 122 patients (79.2%) were dead, 112 (72.3%) from ovarian cancer, and 32 (20.8%) were alive. All tumors were re-evaluated by a board certified pathologist (KJ) and histological grading performed according to a universal system [16].

Information regarding clinical stage was obtained from the medical charts, following the standardized FIGO classification of tumor staging. Information on residual tumor after surgery was not available. Standard adjuvant therapy was platinum-based chemotherapy, from the 1990s given in combination with paclitaxel. Ethical permission was obtained from the Ethics Committee at Lund University. Study design, methodological and technical considerations, as well as data presentation were based on the REMARK criteria [17]. Tissue microarrays (TMAs) had been constructed as previously described [11], whereby two 1.0 mm cores were taken from viable, non-necrotic primary tumor areas. Fallopian tubes with no evidence of histological disease were also sampled from 38 cases. For immunohistochemical analysis, 4 μm TMA-sections were automatically pre-treated using the PT-link system (DAKO, Glostrup, Denmark) and then stained in an Autostainer Plus (DAKO, Glostrup, Denmark) with a monoclonal anti-SATB1 antibody (Clone EPR3895, Epitomics, Burlingame, CA, USA) diluted 1:100. The specificity of the antibody towards SATB1 has been demonstrated previously [8]. The estimated percentage of cells with nuclear SATB1 expression was recorded, as well as the predominant nuclear intensity, denoted as negative (0), weak (1), moderate (2) or strong (3). A combined nuclear score was constructed by multiplying fraction and intensity. Stromal lymphocytes served as positive internal controls and normal colorectal mucosa as negative control [8,9]. Immunohistochemical staining for androgen, estrogen and progesterone receptors (AR, ER and PR), RNA-binding motif protein 3 (RBM3), minichromosome maintenance 3 protein (MCM3), Chek1, Chek2, Ki67 and Dachshund 2 protein (DACH2) was performed as previously described [11-14].

Spearman´s Rho test was used for comparison of SATB1 expression (nuclear score) with clinicopathological and tumour biological factors. Kaplan-Meier analysis and log rank test were applied to illustrate differences in ovarian cancer specific survival (OCSS) and 5-year overall survival (OS) in strata according to negative (0-1%) and positive (>1%) SATB1 expression. Cox regression proportional hazards models were used for estimation of hazard ratios (HRs) for death from ovarian cancer or overall causes within 5 years according to negative and positive SATB1 expression in both uni- and multivariable analysis in high-grade tumours, adjusted for age and clinical stage. All calculations were performed using IBM SPSS Statistics Version 20 (SPSS Inc, Chicago, IL). All statistical tests were two-sided and a p value < 0.05 was considered statistically significant.

## Results

**Figure 1:**
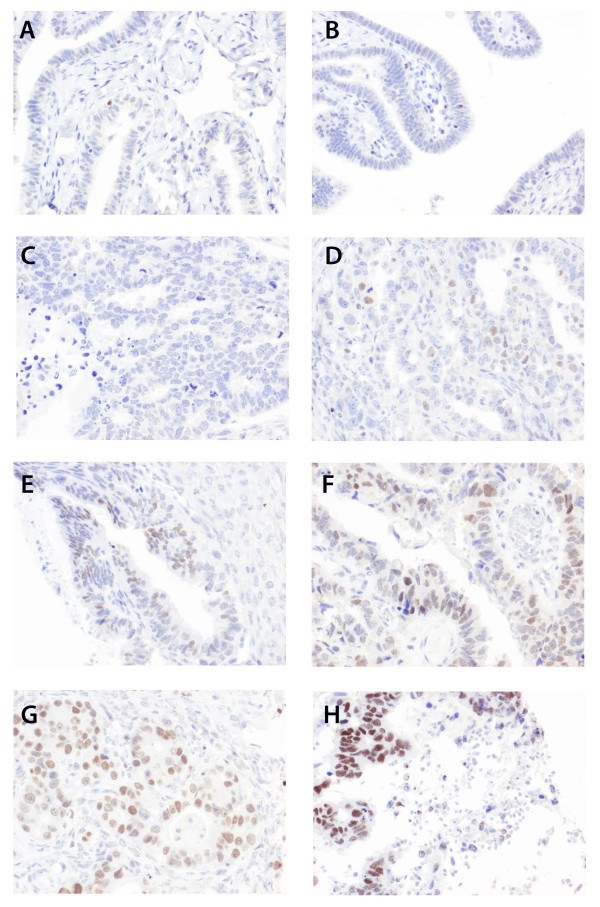
**Immunohistochemical images of SATB1 staining in fallopian tubes and ovarian cancer.** Images (20X magnification) demonstrating negative immunohistochemical expression of SATB1 in (**A**, **B**) fallopian tubes, and different fractions and staining intensities in EOC ranging from (**C**) negative, (**D**-**G**) weak to moderate intensity in increasing fractions and (**H**) strong intensity in the majority of tumour cells.

**Figure 2:**
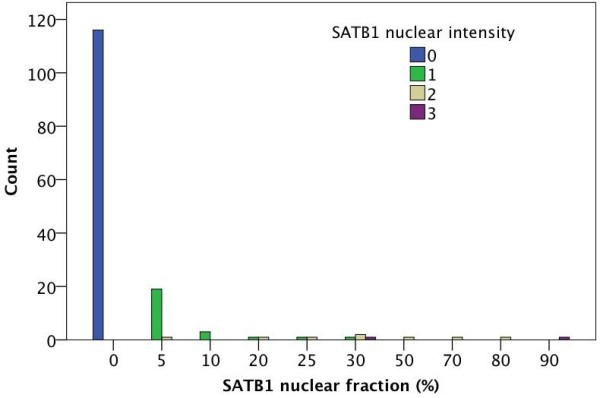
**Distribution of SATB1 staining in primary tumours.** Bar chart visualizing the relationship of nuclear SATB1 staining intensity with the estimated proportion of tumour cells expressing SATB1.

In 32/38 (84.2%) evaluable cases of benign-appearing fallopian tubal epithelium, no or very low levels of SATB1 expression could be detected (Figure 1 A, B). In primary EOC, positive SATB1 expression was denoted in 35/151 (23.2%) evaluable cases, predominantly in fractions <50% and intensities ranging from weak to moderate (Figure 1 C-H and Figure 2), and always exceeding tubal expression. The associations of SATB1 expression with established clinicpathological factors and investigative markers is shown in Table 1. SATB1 expression was significantly associated with lower histological grade (Spearman´s Rho = -0.22, p = 0.006) but not with age or clinical stage. SATB1 expression did not differ by histological subtype (data not shown). There was no significant correlation between SATB1 expression and expression of AR, ER, PR, Ki67, Chek1, Chek2, pChek2 or RBM3. A positive correlation was seen between SATB1 and DACH2 expression (R = 0.28, p = 0.001), pChek1 (R = 0.26, p = 0.001), and MCM3 expression (R = 0.17, p = 0.042).

**Table 1:** Associations of SATB1 expression with clinicopathological and molecular parameters

**Factor**	**SATB1 expresssion**
**Age**	
R	0.044
p	0.603
n	143
**Differentiation grade**	
R	−0.223
p	0.006**
n	151
**Clinical stage**	
R	−0.039
p	0.651
n	139
**Ki67**	
R	−0.025
p	0.764
n	149
**AR**	
R	0.003
p	0.973
n	151
**ER**	
R	−0.072
p	0.388
n	145
**PR**	
R	0.060
p	0.469
n	146
**DACH2**	
R	0.280
p	0.001**
n	143
**RBM3**	
R	−0.092
p	0.263
n	149
**Chek1**	
R	0.079
p	0.353
n	139
**pChek1**	
R	0.260
p	0.002*
n	139
**Chek2**	
R	0.079
p	0.344
n	144
**pChek2**	
R	0.130
p	0.125
n	141
**MCM3**	
R	0.172
p	0.042*
n	140

R = Spearman´s correlation coefficient, p = p-value, n = number of cases available for analysis. ER = estrogen receptor, PR = progesterone receptor, AR = Androgen receptor. *significance at 5% level, ** significance at 1% level. The analyses are based on multipliers of staining intensity and fraction (nuclear score) for expression of SATB1, DACH2, RBM3, Chek1, Chek2 and MCM3 and categories of nuclear fraction for expression of Ki67, AR, ER, and PR.

**Figure 3:**
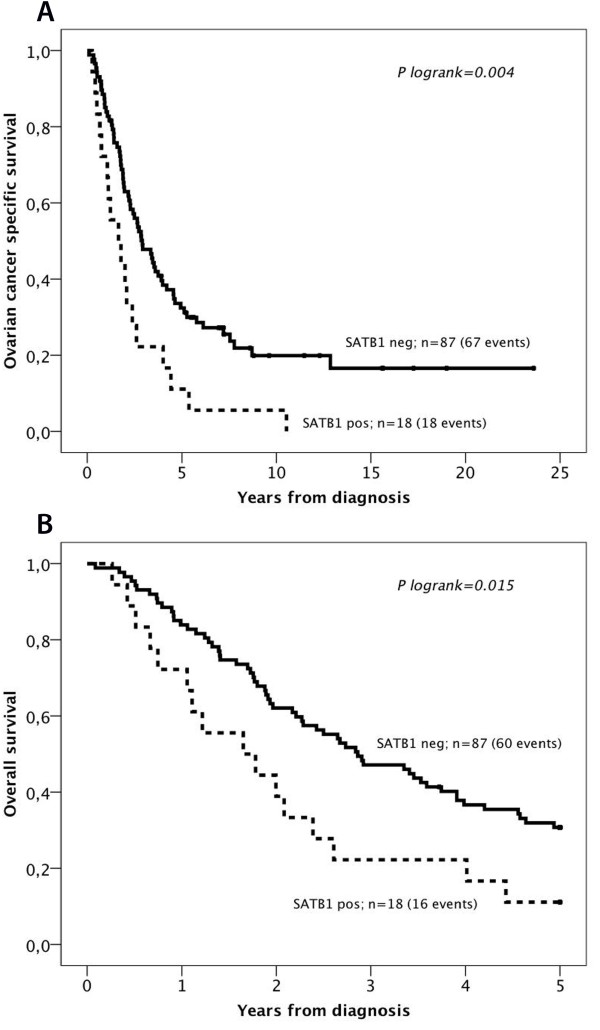
**Kaplan-Meier estimates of survival according to SATB1 expression in patients with high-grade tumours.** Kaplan Meier analysis of (**A**) ovarian cancer specific and (**B**) 5-year overall survival in strata according to negative and positive SATB1 expression in patients with high-grade tumours (n = 105).

Kaplan-Meier analysis revealed no significant association of SATB1 expression with OCSS or OS in the full cohort (data not shown) but stratified analysis according to tumour grade revealed that positive SATB1 expression was a significant factor of poor prognosis in high grade tumours (n = 105), regardless of histological subtype (logrank p = 0.004 for OCSS and logrank p = 0.015 for 5-year OS, (Figure 3 A-B). These associations were confirmed in univariable and multivariable Cox regression analysis, adjusted for age and clinical stage (Table 2). SATB1 expression was not prognostic in low-grade tumours or in subgroups according to histological type (data not shown).

**Table 2:** Relative risks of death from ovarian cancer and overall death according to SATB1 expression in patients with high-grade tumours

	**Ovarian cancer specific survival**			**5-year overall survival**		
	**HR(95%CI)**	***p-value***	***n(events)***	**HR(95%CI)**	***p-value***	***n(events)***
		***Univariable***			***Univariable***	
SATB1 neg	1.00	0.005	87(67)	1,00	0.017	87(60)
SATB1 pos	2.14(1.26-3.62)		18(18)	1.96(1.13-3.42)		18(16)
		**Multivariable**			**Multivariable**	
SATB1 neg	1.00	0.009	82(62)	1,00	0.022	82(55)
SATB1 pos	2.20(1.21-3.99)		14(14)	2.06(1.11-3.81)		14(13)

Cox uni- and multivariable analysis of relative risks of death from ovarian cancer and overall death according to SATB1 expression in patients with high-grade carcinomas, irrespective of histological subtype (n = 105). HR = Hazard ratio. Multivariable analysis included adjustment for age and clinical stage (1–2 vs 3–4).

## Discussion

The results from this study demonstrate that SATB1 expression is an independent factor of poor prognosis in high grade ovarian carcinoma, regardless of histological subtype. These findings are in line with previous studies on the prognostic value of SATB1 expression in EOC and several other cancer forms [3-7,10] and thus further support the notion that the regulatory activities of SATB1 in cancer preferentially seem to confer a more malignant phenotype [18]. In the present study, SATB1 expression was found to be up-regulated in EOC compared to tubal epithelium, from which a proportion of serous carcinomas are though to arise [19]. These findings further underline a role for SATB1 in ovarian carcinogenesis. No associations were found between SATB1 expression and expression of hormone receptors. In breast cancer, one study found SATB1 mRNA expression levels to be higher in ER negative compared to ER positive tumours [20] and in another study, high SATB1 mRNA expression was found to correlate with an improved prognosis in ER positive but not in ER negative tumours, although this did not remain significant in multivariable analysis [21]. Notably, both of these studies relied on gene expression data only [20,21] and none could confirm the negative prognostic value of SATB1 expression in breast cancer demonstrated by Han et al. [3], who found immunohistochemical SATB1 expression to be an independent factor of poor prognosis [3]. Compared to gene expression analyses, immunohistochemistry has some advantages in biomarker studies since it allows for quantitative assessment of proteins in a morphological and subcellular context, which might have important prognostic implications. SATB1 is not only expressed in tumour cell nuclei, but also in stromal lymphocytes, serving as internal staining controls, and our results demonstrate that the prognostic impact of SATB1 was evident even at low levels of expression. These findings are consistent with the study by Han et al., where immunohistochemical expression of SATB1 was denoted as being weak in the majority of the analysed breast cancer samples, and it was demonstrated that even low levels of SATB1 correlated with poor prognosis [3].

A limitation to the here analyzed cohort is the lack of information on residual tumour after surgery, and therefore, the prognostic value of SATB1 expression in EOC should be confirmed in studies on tumours for which this information is available. The inverse correlation between SATB1 expression and histological grade might contribute to the lack of prognostic value for SATB1 in the full cohort. Xiang et al. found no correlation between SATB1 expression and grade, but a positive association with clinical stage [10].

Notably, the heterogeneity among EOC is not only reflected in the occurence of different histological subtypes but also in their mode of progression, i.e. through a stepwise mutation process (low-grade pathway) or through greater genetic instability (high-grade pathway) [22]. Therefore, despite the lack of a more thorough molecular classification of the here studied tumours, and the use of a universal rather than subtype-specific grading system, our results indicate that the tumour-promoting effects of SATB1 expression in EOC differs according to mutational status and genetic stability of the tumours. The associations of SATB1 with expression of MCM3 and phosphorylated Chek1 imply a link between SATB1 and maintenance of DNA integrity [13], and expression of both MCM3 and DACH2 has previously been demonstrated to correlate with poor prognosis in EOC [13,14].

## Conclusions

This study provides further evidence of important regulatory functions of SATB1 in ovarian carcinogenesis and progression, and demonstrate SATB1 expression to be an independent factor of poor prognosis in high-grade tumours. Future studies should address the mechanistic basis for these functions in the context of molecular aberrations and chemotherapy response.

## Abbreviations

EOC: Epithelial ovarian cancer; SATB1: Special AT-rich sequence binding protein 1; AR: Androgen receptor; ER: Estrogen receptor; PR: Progesterone receptor; RBM3: RNA-binding motif protein 3; MCM3: Minichromosome maintenance 3 protein; DACH2: Dachshund 2 protein; NS: Nuclear score; OCSS: Ovarian cancer specific survival; OS: Overall survival.

## Competing interests

The authors declare that they have no competing interests.

## Authors' contributions

BN carried out the immunohistochemical stainings and evaluation, performed statistical analysis, and drafted the manuscript. CH carried out the immunohistochemical evaluation and helped to draft the manuscript. MU participated in the design of the study and provided technical assistance. KJ conceived of the study and participated in its design and coordination and helped to draft the manuscript. All authors read and approved the final manuscript.
